# Polygenic risk discriminates Lewy body dementia from Alzheimer's disease

**DOI:** 10.1002/alz.14381

**Published:** 2025-01-24

**Authors:** Anna McKeever, Peter Swann, Maura Malpetti, Paul C. Donaghy, Alan Thomas, Elijah Mak, Stephen F. Carter, Jerry H. K. Tan, Young T. Hong, Tim D. Fryer, Amanda Heslegrave, Henrik Zetterberg, Li Su, Leonidas Chouliaras, James B. Rowe, John T. O'Brien

**Affiliations:** ^1^ Department of Psychiatry University of Cambridge School of Clinical Medicine Cambridge Biomedical Campus Cambridge UK; ^2^ Cambridgeshire and Peterborough NHS Foundation Trust Cambridge UK; ^3^ Department of Clinical Neurosciences University of Cambridge School of Clinical Medicine Cambridge Biomedical Campus Cambridge UK; ^4^ Cambridge University Hospitals NHS Foundation Trust Cambridge UK; ^5^ Translational and Clinical Research Institute Newcastle University Newcastle upon Tyne UK; ^6^ Wolfson Brain Imaging Centre Department of Clinical Neurosciences University of Cambridge Cambridge Biomedical Campus Cambridge UK; ^7^ Department of Neurodegenerative Disease UCL Queen Square Institute of Neurology London UK; ^8^ Dementia Research Institute UCL London UK; ^9^ Department of Psychiatry and Neurochemistry Institute of Neuroscience and Physiology The Sahlgrenska Academy University of Gothenburg Gothenburg Sweden; ^10^ Clinical Neurochemistry Laboratory Sahlgrenska University Hospital Gothenburg Sweden; ^11^ Hong Kong Center for Neurodegenerative Diseases Hong Kong Science Park Hong Kong Hong Kong; ^12^ Wisconsin Alzheimer's Disease Research Center University of Wisconsin School of Medicine and Public Health University of Wisconsin‐Madison Madison Wisconsin USA; ^13^ Neuroscience Institute University of Sheffield Sheffield UK; ^14^ Specialist Dementia and Frailty Service Essex Partnership University NHS Foundation Trust Essex UK

**Keywords:** Alzheimer's disease, Lewy body dementia, plasma biomarkers, plasma phosphorylated tau, polygenic risk score

## Abstract

**INTRODUCTION:**

Lewy body dementia (LBD) shares genetic risk factors with Alzheimer's disease (AD), including apolipoprotein E *(APOE*), but is distinguishable at the genome‐wide level. Polygenic risk scores (PRS) may therefore improve diagnostic classification.

**METHODS:**

We assessed diagnostic classification using AD‐PRS excluding *APOE* (AD‐PRS_no_ *_APOE_*), *APOE* risk score (*APOE*‐RS), and plasma phosphorylated tau 181 (p‐tau181), in 83 participants with LBD, 27 with positron emission tomography amyloid beta (Aβ)positive mild cognitive impairment or AD (MCI+/AD), and 57 controls.

**RESULTS:**

Together AD‐PRS_no_ *_APOE_* and *APOE*‐RS performed similarly to p‐tau181 in discriminating MCI+/AD from controls (area under the curve 76% vs. 79%) and LBD (71% vs. 72%). In LBD, Aβ positivity was significantly associated with *APOE‐*RS, but not with AD‐PRS_no_ *_APOE_*, or p‐tau181. Combining AD‐PRS_no_ *_APOE_*, *APOE‐*RS, and p‐tau181 improved the discrimination of MCI+/AD from controls (81%) and LBD (75%), and the detection of Aβ in LBD (82%).

**DISCUSSION:**

Aβ deposition in LBD was associated with *APOE*, while MCI+/AD was also associated with AD‐PRS beyond *APOE*. AD‐PRS explains phenotypic variance not captured by *APOE* or p‐tau181.

**Highlights:**

- We investigated Alzheimer's disease (AD) polygenic risk score (PRS), apolipoprotein E (*APOE*), and plasma phosphorylated tau 181 (p‐tau181) to classify AD and Lewy body dementia (LBD).
- AD‐PRS with *APOE* achieved similar classification accuracy to p‐tau181.
- AD‐PRS without *APOE* significantly contributed to discriminating AD from LBD.
- Amyloid beta positivity in LBD was associated with *APOE* but not AD‐PRS without *APOE* or p‐tau181.
- Combining AD‐PRS, *APOE*, and p‐tau181 improved diagnostic classification accuracy.

## 1 BACKGROUND

The accurate diagnosis of dementia syndromes such as Alzheimer's disease (AD) and Lewy body dementia (LBD) remains challenging in early stages and for cases of mixed LB/AD pathology. LBD comprises dementia with Lewy bodies^1^ and Parkinson's disease (PD) dementia,^2^ the difference based on the relative timing of onset (or presence) of a movement disorder relative to cognitive decline. Co‐morbid amyloid beta (Aβ) and Lewy body pathology is common: using positron emission tomography (PET) Aβ imaging, abnormal Aβ deposition is detected in approximately 50% of people with LBD,^3^, ^4^ and using real‐time quaking‐induced conversion, α‐synuclein pathology is detected in approximately 30% of healthy older adults with Aβ pathology.^5^ Such co‐pathology is not discernible clinically without the use of biomarkers and is associated with poorer clinical outcomes.^4^, ^6^, ^7^, ^8^ Efforts to improve diagnostic accuracy using minimally invasive tests are expected to benefit clinical practice, allowing earlier stratification for treatment, clinical trials, and informing prognosis.^9^

The risk of both AD and LBD is partially heritable, at approximately 60% to 80% for AD (from twin studies)^10^ and up to 60% for dementia with Lewy bodies (DLB) (using single nucleotide polymorphism [SNP] heritability).^11^ Genome‐wide association studies (GWAS) have reported > 90 loci associated with late‐onset AD, mostly with small effect sizes compared to the strongest genetic risk factor, apolipoprotein E (*APOE*) ɛ4.^12^ In comparison, GWAS of DLB^13^ and LBD^14^ have been smaller, which limits their statistical power to detect common variants with small effects on disease risk. The largest LBD GWAS to date (including 2591 cases of clinically probable or autopsy‐confirmed LBD) identified five loci reaching genome‐wide statistical significance, which are also associated with either AD or PD.^14^ *APOE* ɛ4 has been strongly associated with LBD in neuropathological studies^15^ and GWAS.^14^, ^16^ While this could be partially attributed to AD co‐pathology, several studies suggest *APOE* ɛ4 drives α‐synuclein pathology independently of AD.^17^, ^18^

LBD shares a proportion of risk variants with AD and PD but is distinguishable at the whole genome level.^11^, ^19^ There is also evidence indicating genetically distinct subgroups of LBD depending on sex^20^ and the extent of AD co‐pathology.^17^, ^21^, ^22^ Polygenic risk scores (PRS) quantify genetic risk conferred by multiple loci, giving a single value estimate of genome‐wide liability.^23^ Information pertaining to individuals’ genetic risk of AD and LBD has potential to support clinicians in refining differential diagnoses and improve participant selection in clinical research. *APOE* ɛ4 alone is not sufficiently sensitive or specific for AD^24^ and further studies are needed on applications of PRS (which are typically composed of many genetic variants with small effects on disease risk^25^), beyond the classification of AD cases and controls.

Several blood‐based biomarkers have been developed as diagnostic and monitoring tools for the neurodegenerative diseases that cause dementia.^26^ High‐sensitivity assays for plasma phosphorylated tau (p‐tau) protein achieve sensitivity and specificity > 90% for AD pathology^27^, ^28^ and may be altered when there is AD co‐pathology in LBD (as shown previously in a sample from this cohort^29^ and independent studies^30^). They may also predict response to future disease‐modifying treatments in LBD.^31^ Due to the high prevalence of concurrent Aβ pathology in LBD, blood‐based biomarkers for AD perform less well discriminating AD from LBD (including plasma p‐tau and Aβ assays, which are generally less accurate than p‐tau).^32^, ^33^, ^34^ Assays for misfolded α‐synuclein and L‐amino acid decarboxylase (DDC) in cerebrospinal fluid (CSF) and peripheral tissues are being developed, with early reports of high sensitivity and specificity for Lewy body disorders.^35^, ^36^, ^37^

Combining phenotype with genotype has potential to improve clinical decision making. Addition of *APOE* ε4 status or AD‐PRS to AD plasma biomarkers modestly improved the discrimination of AD cases from controls.^27^, ^38^, ^39^, ^40^, ^41^ However, this approach has not been tested for discriminating AD from LBD, which is clinically more challenging and less accurate  using current plasma p‐tau assays.^32^ We investigated the accuracy of AD‐PRS in classifying AD and LBD, and AD‐PRS effects on Aβ deposition in LBD. We model *APOE* and non‐*APOE* effects separately with the hypothesis that a combination of AD‐PRS with plasma p‐tau improves classification compared to p‐tau alone.

## 2 METHODS

We included 167 participants, 83 with LBD (78 probable DLB diagnosed using international consensus criteria^6^ and five PD dementia diagnosed using the Movement Disorders Society clinical diagnostic criteria^2^), 27 with AD dementia (according to the National Institute on Aging Alzheimer's Association criteria,^42^ *n* = 16) or PET Aβ positive mild cognitive impairment (MCI PET Aβ+, defined as objective cognitive impairment without impairment in activities of daily living, and PET Aβ positivity,^43^ *n* = 11), and 57 healthy controls. Participants with AD and MCI PET Aβ+ were considered asa single disease group, of varying clinical stages (MCI+/AD). Full details of recruitment, diagnostic criteria, and clinical and neuroimaging findings have been published previously.^4^, ^32^, ^44^ As part of their diagnostic work‐up, 36 participants with DLB had abnormal FP‐CIT scans, the others were diagnosed on the basis of core clinical features.^6^ Participants were recruited from multiple sites including memory clinics and services in East Anglia and the North of England, volunteer registers including those held locally and the national Join Dementia Research volunteer register, and participants’ healthy partners. Exclusion criteria such as acute infection, severe physical illness, major concurrent psychiatric disorder, and history of other significant neurological disease were applied. Cognitive and neuropsychiatric assessments were completed at baseline, including the Addenbrooke's Cognitive Examination Revised (ACE‐R), as described elsewhere.^32^

**RESEARCH IN CONTEXT**

1. **Systematic review**: Plasma phosphorylated tau 181 (p‐tau181) classifies Alzheimer's disease (AD) from controls with high accuracy, with modest improvements from adding apolipoprotein E (*APOE*). Discrimination between AD and Lewy body dementia (LBD) is less accurate, likely due to common amyloid beta (Aβ) co‐pathology in LBD. *APOE* is a risk locus for AD and LBD (and associated with Aβ pathology), but they have different polygenic risk profiles (PRS).
2. **Interpretation**: AD‐PRS with *APOE* achieved similar accuracy to p‐tau181 in classifying AD from LBD. Non‐*APOE* effects were significant, with a similar effect size to *APOE* and p‐tau181. In LBD, Aβ positivity was associated with *APOE*, but not AD‐PRS beyond *APOE*, or p‐tau181. A combination of AD‐PRS, *APOE*, and p‐tau181 improved the classification of AD from controls, LBD, and the detection of Aβ in LBD.
3. **Future directions**: AD‐PRS may be useful to refine the diagnostic framework. Future studies should also include emerging LBD‐specific biomarkers. Studies in larger cohorts could optimize AD‐PRS for discriminating LBD from AD.

### 2.1 Genotyping and polygenic risk scores

Genotyping was performed at two centers using the same methodology, with the Illumina OmniExpress‐24 v1.3 micro‐array.^20^ *APOE* genotype (ε2, ε3, ε4) is defined by two SNPs: rs429358 and rs7412. *APOE* was not genotyped directly, rs7412 was derived from the micro‐array, and rs429358 was imputed using the Haplotype Reference Consortium reference panel.^45^ Details of sample collection, processing of genetic material, and quality control are provided in the Supplementary Material.

AD‐PRS was generated using the clumping and thresholding method, weighted with effect sizes from the largest available case–control AD GWAS.^46^, ^47^ There was no overlap between our sample and the GWAS used as the reference for SNP weights. Modeling *APOE* effects separately improves discrimination of AD cases from controls^47^ and permits investigation of *APOE* and non‐*APOE* effects. Therefore, we calculated AD‐PRS_no_ *_APOE_* by removing SNPs in the *APOE* region (chr19:44.4–46.5 Mb). Primary analyses used a *P* value threshold (pT) of < 0.1 for including SNPs, as this optimizes the prediction of AD cases versus controls when *APOE* effects are modeled separately.^47^ Sensitivity analyses included AD‐PRS_no_ *_APOE_* at pT < 5 × 10^−8^ (the threshold for genome‐wide statistical significance) and pT < 1 × 10^−5^ (previously associated with AD and mixed AD in neuropathological cohorts^48^ and supported by studies suggesting AD has an oligogenic rather than polygenic architecture^49^). *APOE* effects were modeled as a separate *APOE* risk score (*APOE*‐RS), comprising *APOE* ε2 + *APOE* ε4 count, weighted with effect sizes from the same AD case–control GWAS.^46^, ^47^ To ease interpretation, genetic risk scores were standardized within the sample (using the mean and standard deviation of the whole sample). The number of SNPs in AD‐PRS_no_ *_APOE_* is shown in Table S1 in supporting information.

### 2.2 PET Aβ

PET imaging with ligands binding Aβ ([11C] Pittsburgh compound B [PiB] or [18F]‐florbetapir) was performed at baseline for 51 people with LBD and 11 people with MCI (MCI PET Aβ+). As described previously, [11C] PiB PET images were obtained for 36 participants (25 LBD and 11 MCI PET Aβ+) at one site (Wolfson Brain Imaging Centre, Cambridge, UK) using a GE Advance PET scanner (GE Healthcare) or GE Discovery 690 PET/CT, over 30 minutes starting 40 minutes post injection of a 550 MBq PiB bolus, using a transmission scan or low‐dose computed tomography (CT) scan for attenuation correction.^32^ For 26 LBD participants recruited at the other site (Newcastle University, Newcastle, UK), images were obtained using a Siemens Biograph‐40 PET‐CT scanner, over 15 minutes commencing 30 to 50 minutes after injection of 370 MBq [18F]‐florbetapir, with attenuation correction using CT images. PET data were co‐registered with 3T T1‐weighted structural magnetic resonance imaging as described previously.^29^, ^32^, ^44^

The Centiloid (CL) scale^50^, ^51^ was used to allow amyloid PET comparisons between different study sites, tracers ([11C]PiB and [18F]‐florbetapir), and methodology. PET Aβ positivity was classified as > 19 CL; 19 CL has been shown to be a tipping point at which Aβ pathology reliably worsens over time, independent of cognitive performance.^43^, ^51^ The Cambridge [11C]PiB standardized uptake value ratio (SUVR) data were acquired for two different studies and converted to CL using a standard^50^ and non‐standard method.^52^ The Newcastle [18F]florbetapir SUVR data were converted to CL using a previously published equation.^53^

### 2.3 Plasma biomarkers

Analysis of plasma biomarkers in this cohort has been published previously.^32^ Plasma p‐tau181 was selected for the primary analysis in a subsample with available genotyping, due to evidence of superior sensitivity and specificity for AD over the other plasma biomarkers that were available for this sample at the time of this analysis (the ratio of Aβ_42_ to Aβ_40_ [Aβ_42/40_], glial fibrillar acidic protein [GFAP], and neurofilament light [NfL]).^27^, ^32^ Subsequently, studies comparing different p‐tau epitopes have shown earlier and stronger associations with AD pathology using p‐tau217 and p‐tau231.^54^ Secondary analyses also included Aβ_42/40_, GFAP, and NfL. Blood biomarkers were quantified at the UK Dementia Research Institute biomarker laboratory using the Quanterix Simoa p‐tau181 assay V2 and the Quanterix Simoa Human Neurology 4‐Plex E assay for Aβ_40_, Aβ_42_, GFAP, and NfL, using Simoa‐HD1 as per the manufacturer's protocol (Quanterix Corp),^55^ as described elsewhere^32^ and in the Supplementary Material. Values were log transformed to achieve near normal distributions and standardized (using the mean and standard deviation of the whole sample) to improve interpretation of odds ratios.

### 2.4 Statistical analysis

Analyses were performed using RStudio 2021.09.0 Build 351 and JASP 0.18.1. Baseline demographics were compared between diagnostic groups (Control, LBD, and MCI+/AD) using one‐way analysis of variance (ANOVA; for age), Kruskal–Wallis (for education and ACE‐R score, which were not normally distributed), and *χ* ^2^ (for sex and *APOE* ε4 status). Baseline demographics in LBD PET Aβ− and LBD PET Aβ+ were compared using independent sample *t* tests (for age and ACE‐R score), Mann–Whitney *U* (for education, which was not normally distributed), Fisher exact test (for sex), and *χ* ^2^ (for *APOE* ε4 status). Binary logistic regression was used to predict diagnosis from AD‐PRS_no_ *_APOE_*, *APOE*‐RS, and log p‐tau181, controlling for age and sex. Classification accuracy was assessed using receiver operating characteristic (ROC) analyses, also corrected for age and sex, using the pROC package in R. ANOVA and Akaike information criterion with correction for sample size (AICc, calculated using the R package MuMIn) were used to compare model fit and parsimony. Stepwise backward elimination was used to confirm the preferred predictors in classification models, removing variables until there was no further improvement in AICc. Sensitivity analyses investigated whether similar AD‐PRS_no_ *_APOE_* effects were observed using pT < 1 × 10^−5^ and pT < 5 × 10^−8^. We applied the same methods to investigate the effect of adding AD‐PRS_no_ *_APOE_* and *APOE*‐RS to a panel of plasma biomarkers, consisting of log p‐tau181, log Aβ_42/40_, log GFAP, and log NfL.

## 3 RESULTS

Participant characteristics are shown in Table 1. Compared to Controls (but not compared to the MCI+/AD group), the LBD group was older (*P*_Tukey _= 0.001), included more male participants (*P* = 0.011), and had fewer years of education (*P*_Dunn _< 0.001). Both the LBD and MCI+/AD groups performed significantly worse than Controls on cognitive assessment (ACE‐R score, *P*_Dunn _< 0.001), and the LBD group performed significantly worse than MCI+/AD (ACE‐R score, *P*_Dunn _= 0.012). There were more *APOE* ε4 carriers in the MCI+/AD group than both the healthy control (*P* = 0.002) and LBD groups (*P* = 0.011). In keeping with previous studies,^3^, ^4^ among 51 LBD participants for whom PET Aβ status was available, 27 (53%) were Aβ positive (LBD PET Aβ+), and 24 (47%) were Aβ negative (LBD PET Aβ−). The LBD PET Aβ+ group was significantly older than the LBD PET Aβ− group (*P* = 0.027), but there were no significant differences in sex, years of education, or cognitive performance between the LBD PET Aβ+ and LBD PET Aβ− groups. There was a significantly higher proportion of *APOE* ε4 carriers in LBD PET Aβ+ (48%) compared to LBD PET Aβ− (17%; *P* = 0.017).

**TABLE 1:** Demographics, *APOE* ε4 status, AD‐PRS_no_ *_APOE_*, and log p‐tau181.

Diagnosis	Control	LBD	MCI+/AD	Total
*n*	57	83	27	167
Mean age (SD)	70.8 (7.44)	75.3 (6.53)^**,^	73.4 (8.60)	73.5 (7.44)
Sex female (%)	23 (40)	16 (19)*	10 (37)	49 (29)
8	14.1 (3.14)	11.6 (2.94)^***^	13.0 (3.26)	12.7 (3.25)
ACE‐R	94.3 (4.20)	**66.3 (14.9)** ^***^	77.8 (9.99)^***,†^ ^b^	
*n* 0/1/2 *APOE* ε4 alleles (% *APOE* ε4 carrier)	41/13/3 (28)	53/25/5 (36)	9/15/3 (63)^**,†^ ^b^	103/53/11 (38)
AD‐PRS_no_ *_APOE_ z* score	−0.101 (1.09)	−0.038 (0.891)	**0.332 (1.10)** ^d^, ^e^	0 (1)
Log p‐tau181 *z* score	−0.419 (0.838)	0.107 (0.995)	**0.554 (1.01)** ^f^, ^g^	0 (1)

	LBD PET Aβ−	LBD PET Aβ+	Total
*n*	24	27	51
Mean age (SD)	72.1 (5.97)	**76.0 (6.33)^‡^**	74.2 (6.42)
Sex female (%)	3 (12.5)	7 (26)	10 (20)
Years of education (SD)	11.8 (2.81)	11.0 (3.25)	11.4 (3.05)
ACE‐R	69.9 (13.0)	65.0 (16.3)	67.3 (14.9)
*n* 0/1/2 *APOE* ε4 alleles (% *APOE* ε4 carrier)	20/4/0 (17)	**14/12/1 (48) ‡**	34/16/1 (33)
AD‐PRS_no_ *_APOE_ z*‐score	0.147 (0.636)	−0.187 (1.07)	−0.030 (0.901)
Log p‐tau181 *z*‐score	−0.254 (1.14)	0.243 (0.817)	0.009 (1.01)

*Note*: The LBD group was significantly older, included more males, and had fewer years of education than Controls. ACE‐R score was significantly lower in LBD and MCI+/AD than Controls, and significantly lower in LBD than MCI+/AD. There was a significantly higher proportion of *APOE* ε4 carriers in the MCI+/AD group than both the Control and LBD groups. In binary logistic regression controlling for age, sex, and *APOE* risk score, AD‐PRS_no_ *_APOE_* was higher in MCI+/AD than both the Control and LBD groups. In binary logistic regression controlling for age and sex, log p‐tau181 was higher in MCI+/AD than in both the Control and LBD groups. The LBD PET Aβ+ group was significantly older and included more *APOE* ε4 carriers than LBD PET Aβ−.

Abbreviations: ACE‐R, Addenbrooke's Cognitive Examination Revised version; AD‐PRS_no_ *_APOE_*, Alzheimer's disease polygenic risk score excluding the apolipoprotein E locus; *APOE*, apolipoprotein E; LBD, Lewy body dementia; LBD PET Aβ−, Lewy body dementia, amyloid positron emission tomography negative; LBD PET Aβ+, Lewy body dementia, amyloid positron emission tomography positive; MCI+/AD, positron emission tomography amyloid beta positive mild cognitive impairment/Alzheimer's disease dementia; p‐tau, phosphorylated tau; SD, standard deviation

^a^ Significant difference from Control, *P* < 0.001 (***), *P* < 0.01 (**), *P* < 0.05 (*).

^b^ Significant difference from LBD, *P* < 0.001 (**^†††^**), *P* < 0.01 (**^††^**), *P* < 0.05 (**^†^**).

^c^ Significant difference from LBD PET Aβ−,*P* < 0.05 (‡).

^d^ Significant difference from Control in binary logistic regression controlling for age, sex, and *APOE* risk score.

^e^ Significant difference from LBD in binary logistic regression controlling for age, sex, and *APOE* risk score.

^f^ Significant difference from Control in binary logistic regression controlling for age and sex.

^g^ Significant difference from LBD in binary logistic regression controlling for age and sex.

### 3.1 Diagnostic classification

**FIGURE 1:**
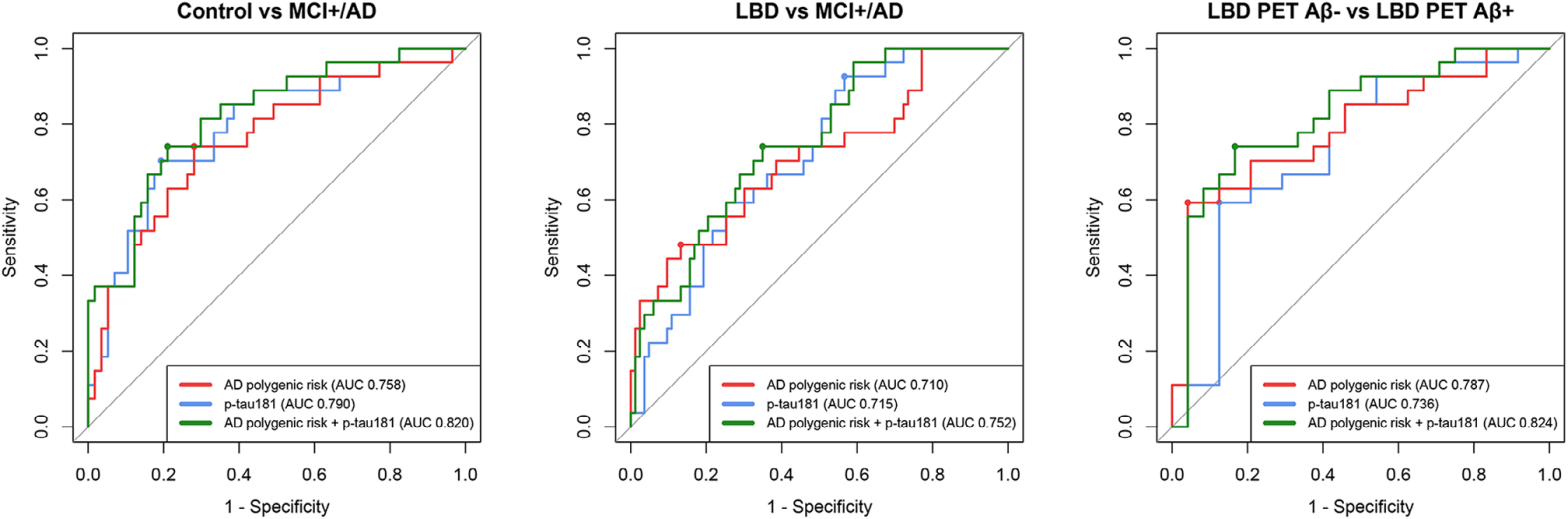
Classification accuracy using AD polygenic risk (AD‐PRS_no_ *_APOE_* and *APOE*‐RS), p‐tau181 (which has also been described in this cohort previously^32^), and a combination of AD polygenic risk (AD‐PRS_no_ *_APOE_* and *APOE*‐RS) and p‐tau181. Age and sex are included in all models as control covariates. Abbreviations: AD polygenic risk, Alzheimer's disease polygenic risk; AD‐PRS_no_ *_APOE_*, Alzheimer's disease polygenic risk score excluding the apolipoprotein E locus; *APOE*, apolipoprotein E; *APOE*‐RS, apolipoprotein E risk score; AUC, area under the curve; LBD, Lewy body dementia; LBD PET Aβ−, Lewy body dementia, amyloid beta positron emission tomography negative; LBD PET Aβ+, Lewy body dementia, amyloid beta positron emission tomography positive; MCI+/AD, positron emission tomography amyloid beta positive mild cognitive impairment / Alzheimer's disease dementia; p‐tau, phosphorylated tau.

AD‐PRS_no_ *_APOE_*, *APOE*‐RS, and p‐tau181 were significantly higher in MCI+/AD than in Controls (see Table 2). When AD‐PRS_no_ *_APOE_*, *APOE*‐RS, and p‐tau181 were included in the classification model together, all remained significantly associated with MCI+/AD, there was a modest improvement in classification accuracy (see Table 2 and Figure 1) and model fit significantly improved compared to the polygenic risk model (ANOVA *P* < 0.001, ΔAICc =  −8.7) and p‐tau181 model (ANOVA *P* = 0.020, ΔAICc = −3.2). After backward elimination, AD‐PRS_no_ *_APOE_* (*P* = 0.037), *APOE*‐RS (*P* = 0.034), and p‐tau181 (*P* < 0.001) were retained, indicating the best trade‐off between fit and parsimony. Trends in AD‐PRS_no_ *_APOE_* were similar across pT < 5 × 10^−8^, pT < 1 × 10^−5^, and pT < 0.1 (see Figure S1 in supporting information), and results of sensitivity analyses (using AD‐PRS_no_ *_APOE_* with pT < 5 × 10^−8^ and pT < 1 × 10^−5^) were consistent (see Tables S2 and S3 in supporting information).

**TABLE 2:** Diagnostic classification. AD‐PRS_no_ *_APOE_*, *APOE*‐RS, and p‐tau181 were significantly higher in MCI+/AD than in both the Control and LBD groups. *APOE*‐RS was significantly higher in LBD PET Aβ+ than in LBD PET Aβ‐.

		Odds ratio [95% CI] (*P* value)						
Diagnosis	Model	AD‐PRS_no_ *_APOE_*	*APOE*‐RS	p‐tau181	Age	Sex	AUC [95% CI]	AICc
Control vs. MCI+/AD	*Polygenic risk*	1.69* [1.05, 2.87] (0.036)	2.32** [1.40, 4.13] (0.002)	–	1.06 [0.992, 1.13] (0.095)	1.45 [0.502, 4.42] (0.499)	0.758 [0.644, 0.872]	100
	*p‐tau181*	**–**	–	3.41*** [1.85, 7.03] (< 0.001)	1.00 [0.932, 1.07] (0.990)	0.579 [0.180, 1.77] (0.343)	0.790 [0.684, 0.896]	94.5
	*Polygenic risk + p‐tau181*	1.79* [1.07, 3.30] (0.038)	1.81* [1.03, 3.34] (0.045)	3.03** [1.55, 6.47] (0.002)	1.01 [0.939, 1.09] (0.783)	0.791 [0.232, 2.65] (0.702)	0.820 [0.723, 0.917]	91.3
LBD vs. MCI+/AD	*Polygenic risk*	1.69* [1.01, 2.90] (0.048)	1.76* [1.12, 2.85] (0.017)	**–**	0.963 [0.899, 1.03] (0.268)	0.440 [0.158, 1.23] (0.113)	0.710 [0.590, 0.830]	119
	*p‐tau181*	**–**	–	1.81* [1.13, 3.01] (0.016)	0.938 [0.875, 1.00] (0.057)	0.333* [0.119, 0.925] (0.034)	0.715 [0.612, 0.819]	120
	*Polygenic risk + p‐tau181*	1.74* [1.04, 3.04] (0.038)	1.62* [1.01, 2.66] (0.049)	1.75* [1.06, 3.00] (0.032)	0.940 [0.873, 1.01] (0.086)	0.382 [0.130, 1.11] (0.075)	0.752 [0.652, 0.853]	117
LBD PET‐Aβ− vs. LBD PET‐Aβ+	*Polygenic risk*	0.579 [0.265, 1.19] (0.142)	2.82* [1.26, 7.50] (0.021)	**–**	1.14* [1.03, 1.29] (0.022)	0.855 [0.129, 5.25] (0.865)	0.787 [0.659, 0.915]	66.6
	*p‐tau181*	**–**	–	1.65 [0.867, 3.50] (0.153)	1.08 [0.981, 1.21] (0.123)	0.515 [0.906, 2.47] (0.419)	0.736 [0.591–0.881]	71.7
	*Polygenic risk* *+ p‐tau181*	0.571 [0.259, 1.18] (0.137)	2.81* [1.21, 7.76] (0.027)	1.63 [0.753, 3.84] (0.232)	1.13* [1.01, 1.28] (0.042)	0.771 [0.112, 5.10] (0.784)	0.824 [0.705, 0.943]	67.7

*Note*: All models include age and sex (Female = 1, Male = 2) as control covariates. AICc quantifies the trade‐off between model fit and parsimony, Δ≥‐2 indicates significant improvement.

Abbreviations: AD‐PRS_no_ *_APOE_*, Alzheimer's disease polygenic risk score excluding the apolipoprotein E locus; AICc, Akaike information criterion with correction for sample size; *APOE*, apolipoprotein E; *APOE*‐RS, apolipoprotein E risk score; AUC, area under receiver operating curve (for the model, including age and sex); CI, confidence interval; LBD, Lewy body dementia; LBD PET Aβ−, Lewy body dementia, amyloid beta positron emission tomography negative; LBD PET Aβ+, Lewy body dementia, amyloid beta positron emission tomography positive; MCI+/AD = amyloid beta positron emission tomography positive mild cognitive impairment / Alzheimer's disease dementia; p‐tau, phosphorylated tau.

^a^ Significant difference between groups*P* < 0.001 (***), *P* < 0.01 (**), *P* < 0.05 (*).

AD‐PRS_no_ *_APOE_*, *APOE*‐RS, and p‐tau181 were also higher in MCI+/AD than LBD, with similar effect sizes, and all remained significant in the full model including polygenic risk and p‐tau181 (see Table 2). Using AD‐PRS_no_ *_APOE_*, *APOE*‐RS, and p‐tau181 together, there was an improvement in classification accuracy (Table 2 and Figure 1) and significant improvement in model fit compared to the polygenic risk model (ANOVA *P* = 0.027, ΔAICc = −2), and p‐tau181 model (ANOVA *P* = 0.026, ΔAICc = −3). All predictors were retained after backward elimination, including age and sex. In sensitivity analyses, results were similar for AD‐PRS_no_ *_APOE_* pT < 5 × 10^−8^ but we did not detect a significant difference in AD‐PRS_no_ *_APOE_* pT < 1 × 10^−5^ between LBD and MCI+/AD (see Tables S2 and S3).

### 3.2 Classification of PET Aβ status in LBD

In LBD participants with available PET Aβ status, LBD PET Aβ+ was associated with higher *APOE*‐RS, but there were no significant differences in AD‐PRS_no_ *_APOE_* or p‐tau181 between LBD PET Aβ− and LBD PET Aβ+ (see Table 2). The addition of AD‐PRS_no_ *_APOE_* and *APOE*‐RS to a classification model including p‐tau181, age, and sex improved classification accuracy (from 74% to 82%, see Table 2 and Figure 1) and significantly improved model fit (ANOVA *P* = 0.011, ΔAICc = −4.0), likely due to significant *APOE* effects. Conversely, the addition of p‐tau181 to the polygenic risk model did not improve model fit (ANOVA *P* = 0.22, ΔAICc = +1.1). Backward elimination showed the optimal balance between model fit and parsimony was achieved from a model including only *APOE*‐RS (*P* = 0.017), AD‐PRS_no_ *_APOE_* (*P* = 0.145), and age (*P* = 0.014). Sensitivity analyses with *AD‐PRS_noAPOE_* pT < 5 × 10^−8^ and pT < 1 × 10^−5^ were overall consistent (see Tables S2 and S3), but only *APOE*‐RS and age were retained in parsimonious models.

### 3.3 Classification using polygenic risk and a panel of plasma biomarkers

The addition of genetic risk scores (AD‐PRS_no_ *_APOE_* and *APOE*‐RS) to a panel of plasma biomarkers (p‐tau181, Aβ_42/40_, GFAP, and NfL), also improved discrimination of LBD from MCI+/AD, and LBD PET Aβ+ from LBD PET Aβ−, in terms of both model fit (*P* = 0.028 and *P* = 0.029, respectively) and classification accuracy (AUC improved from 74% to 78%, and 76% to 84%, respectively, see Table S4 in supporting information). In classifying MCI+/AD and controls, adding genetic risk scores to the plasma biomarker panel did not significantly improve model fit (*P* = 0.078), while classification accuracy improved from 82% to 84%. Using backward elimination to identify the optimal combination of plasma biomarkers and genetic risk factors, both AD‐PRS_no_ *_APOE_* and *APOE*‐RS were retained in the parsimonious models for all diagnostic contrasts (see Table S4).

## 4 DISCUSSION

We confirmed the value of PRS in addition to plasma p‐tau181 or a panel of biomarkers of neurodegeneration in diagnostic classification between healthy adults, people with LBD with and without Aβ co‐pathology, and people with MCI+/AD. In discriminating MCI+/AD from LBD and controls, the addition of AD‐PRS_no_ *_APOE_* and *APOE*‐RS to p‐tau181, age, and sex, resulted in significant improvements in model fit and modest improvements in classification accuracy (AUC). When AD‐PRS_no_ *_APOE_*, *APOE*‐RS, and p‐tau181 were included together, *APOE* effects were less significant. This likely reflects the correlation between *APOE* genotype and p‐tau181, in line with previous studies showing plasma p‐tau levels are strongly related to *APOE*.^40^, ^56^ In classification of MCI+/AD and LBD, effect sizes were similar for AD‐PRS_no_ *_APOE_*, *APOE*‐RS, and p‐tau181. Our results indicate that AD polygenic risk (including beyond the *APOE* locus) explains phenotypic variance in MCI+/AD not captured by p‐tau181. This may be because AD‐PRS_no_ *_APOE_* includes risk variants in multiple AD pathways including immunity, lipid metabolism, tau‐binding proteins, and amyloid precursor protein metabolism.^46^

In the LBD subgroup with available PET Aβ, *APOE*‐RS was the only significant predictor of PET Aβ status. This is in keeping with previous studies (in cohorts of healthy controls, MCI, and AD) showing PET Aβ burden is strongly associated with *APOE* genotype, but AD‐PRS beyond the *APOE* locus explains a low proportion of variance, or effects are non‐significant.^41^, ^57^, ^58^, ^59^, ^60^, ^61^, ^62^ Similarly in a large LBD sample (*n* = 394), excluding *APOE* attenuated an association between AD‐PRS and Thal phase.^22^ Abnormal Aβ deposition is necessary but not sufficient for AD, and variants beyond the *APOE* locus may drive conversion from Aβ positivity to AD.^58^, ^63^ Furthermore, we did not detect a significant difference in p‐tau181 between LBD PET Aβ− and LBD PET Aβ+. Addition of genetic risk scores to the p‐tau181 classification model significantly improved model fit and classification accuracy from 74% to 82%, largely due to *APOE* effects. While our study may have been underpowered to detect small AD‐PRS_no_ *_APOE_* effects relating to Aβ deposition in LBD, our results are in keeping with previous evidence that *APOE* effects are more substantial.^41^, ^57^, ^58^, ^59^, ^60^, ^61^, ^62^ Previous studies have reported differential plasma p‐tau217 and p‐tau181 levels in LBD with and without AD co‐pathology (defined by tau PET status using a temporal region of interest,^30^ CSF Aβ_42/40_,^30^, ^64^ and PiB SUVR^65^). The performance of p‐tau181 in differentiating LBD from MCI+/AD, and LBD PET Aβ− from LBD PET Aβ+ in this sample, may relate to the age and disease stage of the LBD group, variation in the definition of AD co‐pathology and plasma p‐tau assays between studies, and potential clinical misdiagnosis (although clinical consensus criteria provide high sensitivity and specificity for the diagnosis of DLB^66^). While age was included as a covariate in all classification models, older age may adversely affect biomarker discrimination between younger AD cases and older non‐AD participants.^40^, ^67^ A neuropathological study has also reported elevated p‐tau181 in pure LBD compared to other non‐AD dementias 4 years *ante mortem*.^28^ Although plasma p‐tau217 levels accurately predict tau pathology in amyloid‐positive individuals without cognitive impairment,^27^ further studies are needed to clarify the specificity of elevated plasma p‐tau181 (and other isoforms) for AD over other causes of neurodegeneration in the context of amyloid accumulation.

While incorporating PRS in disease classification models can present several challenges,^68^ the PRS information complements dementia biomarkers such as peripheral blood concentration of p‐tau. As a genetic signature, PRS should be less susceptible to acute changes due to environmental factors (e.g., head trauma) and co‐morbidities (e.g., renal failure)^28^, ^69^, and can be obtained from non‐invasive sampling methods. PRS are also stable over individual lifetimes and disease stages. Given previous findings that p‐tau181 is less accurate in MCI^34^ and older age groups,^28^, ^34^ further studies on PRS are needed in these populations. Several factors influence how accurately PRS predict disease phenotypes, including the reliability of the GWAS from which effect sizes are derived. This is partly determined by sample size and accurate assignment to case and control arms of the study.^47^ To calculate AD‐PRS, we used the largest GWAS of clinically diagnosed AD cases (*n* = 94,437) as the references for SNP weights,^46^ though several larger AD GWAS have been published using proxy measures such as family history of dementia.^70^ Most GWAS for AD have been performed in cohorts with European ancestry.^12^ Just as *APOE* effects on AD risk differ according to race, ethnicity, and global population ancestry (e.g., with diminishing effect sizes for the ε4 allele from East Asian, non‐Hispanic White, non‐Hispanic Black, to Hispanic populations^71^), PRS derived using these effect sizes may not accurately predict disease risk in other populations.^72^, ^73^ There is a need to diversify genetic studies in common neurodegenerative dementias (beyond AD and cohorts with European ancestry) and demonstrate whether PRS are transferable to diverse clinical populations.^74^ There is no consensus on the optimal method for calculating AD‐PRS to differentiate AD cases from controls, or non‐AD dementias.^47^, ^49^ However there is strong evidence that special consideration of the *APOE* locus is warranted due to substantial effects on disease risk, association with earlier disease onset, and changes in allele frequency with age.^47^ To minimize multiple testing, our primary analysis used methods previously shown to optimize the prediction of AD cases and controls,^47^ and sensitivity analyses gave similar results (see Supplementary Material). PRS prediction of disease biomarker status (PET Aβ− vs. PET Aβ+) and dementia phenotype (LBD vs. AD) differs from the standard application of PRS in discriminating dementia cases from controls. Larger longitudinal studies should consider optimizing PRS to predict co‐pathology and clinical phenotypes (particularly in early disease stages), potentially using PRS restricted to specific disease pathways^22^ or cell types.^75^ Further studies in LBD are also needed to explore potentially distinct genetic risk profiles for males and females,^20^ and LBD with and without AD co‐pathology.^22^

## 5 CONCLUSIONS

Our results show that addition of an individual's AD‐PRS may improve the classification of AD and LBD using plasma p‐tau181 and help detect Aβ co‐pathology in LBD. Genetic risk scores reflect a component of disease risk. The PRS may form part of a tiered approach to identify high‐risk individuals or support a diagnosis in combination with disease biomarkers for those who require confirmatory tests.^9^, ^27^, ^76^ PRS can also be applied to understand the genetic correlation between LBD and AD.^14^ Our results support previous evidence that AD‐PRS beyond the *APOE* locus explains phenotypic variance in AD not captured by *APOE* or p‐tau181. Our findings are in keeping with studies in cohorts of controls, MCI, and AD, showing PET Aβ positivity is strongly associated with the *APOE* genotype, but the non‐*APOE* AD‐PRS was not associated with PET‐Aβ positivity in this sample of individuals with LBD. Future studies on PRS for neurodegenerative dementias should continue to consider applications beyond predicting AD case–control status.

## CONFLICT OF INTEREST STATEMENT

M.M. has acted as a consultant for Astex Pharmaceuticals. P.C.D. has received grant or academic support from Alzheimer's Research UK, Alzheimer's Society, and received honoraria (paid to institution) for educational presentations for the Lewy Body Academy. H.Z. has served on scientific advisory boards and/or as a consultant for Abbvie, Acumen, Alector, Alzinova, ALZPath, Amylyx, Annexon, Apellis, Artery Therapeutics, AZTherapies, Cognito Therapeutics, CogRx, Denali, Eisai, Merry Life, Nervgen, Novo Nordisk, Optoceutics, Passage Bio, Pinteon Therapeutics, Prothena, Red Abbey Labs, reMYND, Roche, Samumed, Siemens Healthineers, Triplet Therapeutics, and Wave; has given lectures in symposia sponsored by Alzecure, Biogen, Cellectricon, Fujirebio, Lilly, Novo Nordisk, and Roche; and is a co‐founder of Brain Biomarker Solutions in Gothenburg AB (BBS), which is a part of the GU Ventures Incubator Program (outside submitted work). L.S. acted as a Research Strategy Council Member, Alzheimer's Society. L.C. was supported by an NIHR Clinical Lectureship, the Academy of Medical Sciences (SGL019∖1035), the Fernblanc Foundation and Alzheimer's Research UK (ARUK‐PPG2018B‐016), and acted as a Clinical Hub Advisor for the Early Detection of Alzheimer's Disease Initiative (EDoN). J.B.R. served on scientific advisory boards and/or as a consultant for Asceneuron Astex, Astronautx, ClinicalInk, CumulusNeuro, Cerevance Curasen, Eisai, ICG, Invicro, Prevail, and Dementia Mission; has received grant or academic support from Janssen, Lilly, GSK, AstraZeneca, Medical Research Council, NIHR, Wellcome Trust, PSP Association, and Alzheimer's Research UK; royalties from Oxford University Press; and is a trustee of the Guarantors of Brain, Darwin College, and the PSP Association. J.T.O'B. has acted as a consultant for TauRx, Novo Nordisk, Biogen, Roche, Lilly GE Healthcare, and Okwin and received grant or academic support from Avid/ Lilly, Merck, and Alliance Medical. A.T., E.M., S.F.C., J.H.T., Y.T.H., T.D.F., and A.H. have nothing to disclose. Author disclosures are available in the supporting information.

## CONSENT STATEMENT

All participants provided informed consent before participating in this study. This study was approved by NIHR National Research Ethics Service Committees: East of England (13/EE/0104) and North East—Newcastle & North Tyneside (13/NE/0064).

## Supporting information



Supporting Information

Supporting Information

## References

[1] McKeith IG , Galasko D , Kosaka K , et al. Consensus guidelines for the clinical and pathologic diagnosis of dementia with Lewy bodies (DLB): report of the consortium on DLB international workshop. Neurology. 1996;47:1113‐1124. doi:10.1212/wnl.47.5.1113 8909416

[2] Emre M , Aarsland D , Brown R , et al. Clinical diagnostic criteria for dementia associated with Parkinson's disease. Mov Disord. 2007;22:1689‐1837. doi:10.1002/mds.21507 17542011

[3] Ossenkoppele R , Jansen WJ , Rabinovici GD , et al. Prevalence of amyloid PET positivity in dementia syndromes: a meta‐analysis. JAMA. 2015;313:1939‐1949. doi:10.1001/jama.2015.4669 25988463 PMC4517678

[4] Donaghy PC , Firbank MJ , Thomas AJ , et al. Clinical and imaging correlates of amyloid deposition in dementia with Lewy bodies. Mov Disord. 2018;33:1130‐1138. doi:10.1002/mds.27403 29672930 PMC6175485

[5] Palmqvist S , Rossi M , Hall S , et al. Cognitive effects of Lewy body pathology in clinically unimpaired individuals. Nat Med. 2023;29:1971‐1978. doi:10.1038/s41591-023-02450-0 37464059 PMC10427420

[6] McKeith IG , Boeve BF , Dickson DW , et al. Diagnosis and management of dementia with Lewy bodies: fourth consensus report of the DLB Consortium. Neurology. 2017;89:88‐100. doi:10.1212/WNL.0000000000004058 28592453 PMC5496518

[7] Irwin DJ , Grossman M , Weintraub D , et al. Neuropathological and genetic correlates of survival and dementia onset in synucleinopathies: a retrospective analysis. Lancet Neurol. 2017;16:55‐65. doi:10.1016/S1474-4422(16)30291-5 27979356 PMC5181646

[8] Abdelnour C , van Steenoven I , Londos E , et al. Alzheimer's disease cerebrospinal fluid biomarkers predict cognitive decline in Lewy body dementia. Mov Disord. 2016;31:1203‐1208. doi:10.1002/mds.26668 27296778

[9] Hansson O , Edelmayer RM , Boxer AL , et al. The Alzheimer's Association appropriate use recommendations for blood biomarkers in Alzheimer's disease. Alzheimers Dement. 2022;18:2669‐2686. doi:10.1002/alz.12756 35908251 PMC10087669

[10] Gatz M , Reynolds CA , Fratiglioni L , et al. Role of genes and environments for explaining Alzheimer disease. Arch Gen Psychiatry. 2006;63:168‐174. doi:10.1001/archpsyc.63.2.168 16461860

[11] Guerreiro R , Escott‐Price V , Hernandez DG , et al. Heritability and genetic variance of dementia with Lewy bodies. Neurobiol Dis. 2019;127:492‐501. doi:10.1016/j.nbd.2019.04.004 30953760 PMC6588425

[12] Lambert J‐C , Ramirez A , Grenier‐Boley B , Bellenguez C . Step by step: towards a better understanding of the genetic architecture of Alzheimer's disease. Mol Psychiatry. 2023;28:2716‐2727. doi:10.1038/s41380-023-02076-1 37131074 PMC10615767

[13] Guerreiro R , Ross OA , Kun‐Rodrigues C , et al. Investigating the genetic architecture of dementia with Lewy bodies: a two‐stage genome‐wide association study. Lancet Neurol. 2018;17:64‐74. doi:10.1016/S1474-4422(17)30400-3 29263008 PMC5805394

[14] Chia R , Sabir MS , Bandres‐Ciga S , et al. Genome sequencing analysis identifies new loci associated with Lewy body dementia and provides insights into its genetic architecture. Nat Genet. 2021;53:294‐303. doi:10.1038/s41588-021-00785-3 33589841 PMC7946812

[15] Tsuang D , Leverenz JB , Lopez OL , et al. APOE ε4 increases risk for dementia in pure synucleinopathies. JAMA Neurol. 2013;70:223‐228. doi:10.1001/jamaneurol.2013.600 23407718 PMC3580799

[16] Rongve A , Witoelar A , Ruiz A , et al. GBA and APOE ε4 associate with sporadic dementia with Lewy bodies in European genome wide association study. Sci Rep. 2019;9:7013. doi:10.1038/s41598-019-43458-2 31065058 PMC6504850

[17] Kaivola K , Shah Z , Chia R ; International LBD Genomics Consortium , Scholz SW . Genetic evaluation of dementia with Lewy bodies implicates distinct disease subgroups. Brain. 2022;145:1757‐1762. doi:10.1093/brain/awab402 35381062 PMC9423712

[18] Dickson DW , Heckman MG , Murray ME , et al. APOE ε4 is associated with severity of Lewy body pathology independent of Alzheimer pathology. Neurology. 2018;91:e1182‐e1195. doi:10.1212/WNL.0000000000006212 30143564 PMC6161556

[19] Guerreiro R , Escott‐Price V , Darwent L , et al. Genome‐wide analysis of genetic correlation in dementia with Lewy bodies, Parkinson's and Alzheimer's diseases. Neurobiol Aging. 2016;38:214.e7‐214.e10. doi:10.1016/j.neurobiolaging.2015.10.028 PMC4759606 26643944

[20] Gibbons E , Rongve A , de Rojas I , et al. Identification of a sex‐specific genetic signature in dementia with Lewy bodies: a meta‐analysis of genome‐wide association studies. MedRxiv. 2022:2022.11.22.22282597. doi:10.1101/2022.11.22.22282597

[21] van der Lee SJ , van Steenoven I , van de Beek M , et al. Genetics contributes to concomitant pathology and clinical presentation in dementia with Lewy bodies. J Alzheimers Dis. 2021;83:269‐279. doi:10.3233/JAD-210365 34308904 PMC8461715

[22] Tunold J‐A , Tan MMX , Koga S , et al. Lysosomal polygenic risk is associated with the severity of neuropathology in Lewy body disease. Brain. 2023;146:4077‐4087. doi:10.1093/brain/awad183 37247383 PMC10545498

[23] Choi SW , Mak TS‐H , O'Reilly PF . Tutorial: a guide to performing polygenic risk score analyses. Nat Protoc. 2020;15:2759‐2772. doi:10.1038/s41596-020-0353-1 32709988 PMC7612115

[24] Mayeux R , Saunders AM , Shea S , et al. Utility of the apolipoprotein E genotype in the diagnosis of Alzheimer's disease. N Engl J Med. 1998;338:506‐511. doi:10.1056/NEJM199802193380804 9468467

[25] Torkamani A , Wineinger NE , Topol EJ . The personal and clinical utility of polygenic risk scores. Nat Rev Genet. 2018;19:581‐590. doi:10.1038/s41576-018-0018-x 29789686

[26] Hansson O . Biomarkers for neurodegenerative diseases. Nat Med. 2021;27:954‐963. doi:10.1038/s41591-021-01382-x 34083813

[27] Ashton NJ , Brum WS , Di Molfetta G , et al. Diagnostic accuracy of a plasma phosphorylated tau 217 immunoassay for Alzheimer disease pathology. JAMA Neurol. 2024;81:255‐263. doi:10.1001/jamaneurol.2023.5319 38252443 PMC10804282

[28] Lantero Rodriguez J , Karikari TK , Suárez‐Calvet M , et al. Plasma p‐tau181 accurately predicts Alzheimer's disease pathology at least 8 years prior to post‐mortem and improves the clinical characterisation of cognitive decline. Acta Neuropathol. 2020;140:267‐278. doi:10.1007/s00401-020-02195-x 32720099 PMC7423866

[29] Donaghy PC , Firbank M , Petrides G , et al. The relationship between plasma biomarkers and amyloid PET in dementia with Lewy bodies. Parkinsonism Relat Disord. 2022;101:111‐116. doi:10.1016/j.parkreldis.2022.07.008 35872565

[30] Hall S , Janelidze S , Londos E , et al. Plasma phospho‐tau identifies Alzheimer's co‐pathology in patients with Lewy body disease. Mov Disord. 2021;36:767‐771. doi:10.1002/mds.28370 33285015 PMC8048822

[31] Alam JJ , Maruff P , Doctrow SR , et al. Association of plasma phosphorylated tau with the response to neflamapimod treatment in patients with dementia with Lewy bodies. Neurology. 2023;101:e1708‐e1717. doi:10.1212/WNL.0000000000207755 37657939 PMC10624490

[32] Chouliaras L , Thomas A , Malpetti M , et al. Differential levels of plasma biomarkers of neurodegeneration in Lewy body dementia, Alzheimer's disease, frontotemporal dementia, and progressive supranuclear palsy. J Neurol Neurosurg Psychiatry. 2022;93:651‐658. doi:10.1136/jnnp-2021-327788 35078917 PMC9148982

[33] Thomas AJ , Hamilton CA , Heslegrave A , et al. A longitudinal study of plasma pTau181 in mild cognitive impairment with Lewy bodies and Alzheimer's disease. Mov Disord. 2022;37:1495‐1504. doi:10.1002/mds.28994 35318733 PMC9540809

[34] Hamilton CA , O'Brien J , Heslegrave A , et al. Plasma biomarkers of neurodegeneration in mild cognitive impairment with Lewy bodies. Psychol Med. 2023;53:7865‐7873. doi:10.1017/S0033291723001952 37489795 PMC10755229

[35] Okuzumi A , Hatano T , Matsumoto G , et al. Propagative α‐synuclein seeds as serum biomarkers for synucleinopathies. Nat Med. 2023;29:1448‐1455. doi:10.1038/s41591-023-02358-9 37248302 PMC10287557

[36] Pereira JB , Kumar A , Hall S , et al. DOPA decarboxylase is an emerging biomarker for Parkinsonian disorders including preclinical Lewy body disease. Nat Aging. 2023;3:1201‐1209. doi:10.1038/s43587-023-00478-y 37723208 PMC10570139

[37] Yan S , Jiang C , Janzen A , et al. Neuronally derived extracellular vesicle α‐synuclein as a serum biomarker for individuals at risk of developing Parkinson disease. JAMA Neurol. 2024;81:59‐68. doi:10.1001/jamaneurol.2023.4398 38048087 PMC10696516

[38] Cullen NC , Leuzy A , Janelidze S , et al. Plasma biomarkers of Alzheimer's disease improve prediction of cognitive decline in cognitively unimpaired elderly populations. Nat Commun. 2021;12:3555. doi:10.1038/s41467-021-23746-0 34117234 PMC8196018

[39] Palmqvist S , Tideman P , Cullen N , et al. Prediction of future Alzheimer's disease dementia using plasma phospho‐tau combined with other accessible measures. Nat Med. 2021;27:1034‐1042. doi:10.1038/s41591-021-01348-z 34031605

[40] Stevenson‐Hoare J , Heslegrave A , Leonenko G , et al. Plasma biomarkers and genetics in the diagnosis and prediction of Alzheimer's disease. Brain. 2023;146:690‐699. doi:10.1093/brain/awac128 35383826 PMC9924904

[41] Ramanan VK , Gebre RK , Graff‐Radford J , et al. Genetic risk scores enhance the diagnostic value of plasma biomarkers of brain amyloidosis. Brain. 2023;146:4508‐4519. doi:10.1093/brain/awad196 37279785 PMC10629762

[42] McKhann GM , Knopman DS , Chertkow H , et al. The diagnosis of dementia due to Alzheimer's disease: recommendations from the National Institute on Aging‐Alzheimer's Association workgroups on diagnostic guidelines for Alzheimer's disease. Alzheimers Dement. 2011;7:263‐269. doi:10.1016/j.jalz.2011.03.005 21514250 PMC3312024

[43] Jack CR Jr , Wiste HJ , Weigand SD , et al. Defining imaging biomarker cut points for brain aging and Alzheimer's disease. Alzheimers Dement. 2017;13:205‐216. doi:10.1016/j.jalz.2016.08.005 27697430 PMC5344738

[44] Bevan‐Jones WR , Surendranathan A , Passamonti L , et al. Neuroimaging of Inflammation in Memory and Related Other Disorders (NIMROD) study protocol: a deep phenotyping cohort study of the role of brain inflammation in dementia, depression and other neurological illnesses. BMJ Open. 2017;7:e013187. doi:10.1136/bmjopen-2016-013187 PMC5223666 28064175

[45] McCarthy S , Das S , Kretzschmar W , et al. A reference panel of 64,976 haplotypes for genotype imputation. Nat Genet. 2016;48:1279‐1283. doi:10.1038/ng.3643 27548312 PMC5388176

[46] Kunkle BW , Grenier‐Boley B , Sims R , et al. Genetic meta‐analysis of diagnosed Alzheimer's disease identifies new risk loci and implicates Aβ, tau, immunity and lipid processing. Nat Genet. 2019;51:414‐430. doi:10.1038/s41588-019-0358-2 30820047 PMC6463297

[47] Leonenko G , Baker E , Stevenson‐Hoare J , et al. Identifying individuals with high risk of Alzheimer's disease using polygenic risk scores. Nat Commun. 2021;12:4506. doi:10.1038/s41467-021-24082-z 34301930 PMC8302739

[48] Spencer BE , Jennings RG , Fan CC , Brewer JB . Assessment of genetic risk for improved clinical‐neuropathological correlations. Acta Neuropathol Commun. 2020;8:160. doi:10.1186/s40478-020-01033-1 32912321 PMC7488152

[49] Zhang Q , Sidorenko J , Couvy‐Duchesne B , et al. Risk prediction of late‐onset Alzheimer's disease implies an oligogenic architecture. Nat Commun. 2020;11:4799. doi:10.1038/s41467-020-18534-1 32968074 PMC7511365

[50] Klunk WE , Koeppe RA , Price JC , et al. The Centiloid Project: standardizing quantitative amyloid plaque estimation by PET. Alzheimers Dement. 2015;11:1‐15.e154. doi:10.1016/j.jalz.2014.07.003 25443857 PMC4300247

[51] Pemberton HG , Collij LE , Heeman F , et al. Quantification of amyloid PET for future clinical use: a state‐of‐the‐art review. Eur J Nucl Med Mol Imaging. 2022;49:3508‐3528. doi:10.1007/s00259-022-05784-y 35389071 PMC9308604

[52] Michalowska MM , Herholz K , Hinz R , et al. Evaluation of in vivo staging of amyloid deposition in cognitively unimpaired elderly aged 78‐94. Mol Psychiatry. 2022;27:4335‐4342. doi:10.1038/s41380-022-01685-6 35858992 PMC9718666

[53] Navitsky M , Joshi AD , Kennedy I , et al. Standardization of amyloid quantitation with florbetapir standardized uptake value ratios to the Centiloid scale. Alzheimers Dement. 2018;14:1565‐1571. doi:10.1016/j.jalz.2018.06.1353 30006100

[54] Gonzalez‐Ortiz F , Kac PR , Brum WS , Zetterberg H , Blennow K , Karikari TK . Plasma phospho‐tau in Alzheimer's disease: towards diagnostic and therapeutic trial applications. Mol Neurodegener. 2023;18:18. doi:10.1186/s13024-023-00605-8 36927491 PMC10022272

[55] Rissin DM , Kan CW , Campbell TG , et al. Single‐molecule enzyme‐linked immunosorbent assay detects serum proteins at subfemtomolar concentrations. Nat Biotechnol. 2010;28:595‐599. doi:10.1038/nbt.1641 20495550 PMC2919230

[56] Lord J , Zettergren A , Ashton NJ , et al. A genome‐wide association study of plasma phosphorylated tau181. Neurobiol Aging. 2021;106:304.e1‐304.e3. doi:10.1016/j.neurobiolaging.2021.04.018 34119372

[57] Ge T , Sabuncu MR , Smoller JW , Sperling RA , Mormino EC , Alzheimer's Disease Neuroimaging Initiative . Dissociable influences of APOE epsilon4 and polygenic risk of AD dementia on amyloid and cognition. Neurology. 2018;90:e1605‐e1612. doi:10.1212/WNL.0000000000005415 29592889 PMC5931806

[58] Leonenko G , Shoai M , Bellou E , et al. Genetic risk for alzheimer disease is distinct from genetic risk for amyloid deposition. Ann Neurol. 2019;86:427‐435. doi:10.1002/ana.25530 31199530 PMC6771864

[59] Altmann A , Scelsi MA , Shoai M , et al. A comprehensive analysis of methods for assessing polygenic burden on Alzheimer's disease pathology and risk beyond APOE. Brain Commun. 2020;2:fcz047. doi:10.1093/braincomms/fcz047 32226939 PMC7100005

[60] Darst BF , Koscik RL , Racine AM , et al. Pathway‐specific polygenic risk scores as predictors of amyloid‐β deposition and cognitive function in a sample at increased risk for Alzheimer's disease. J Alzheimers Dis. 2017;55:473‐484. doi:10.3233/JAD-160195 27662287 PMC5123972

[61] Porter T , Burnham SC , Milicic L , et al. Utility of an Alzheimer's disease risk‐weighted polygenic risk score for predicting rates of cognitive decline in preclinical Alzheimer's disease: a prospective longitudinal study. J Alzheimers Dis. 2018;66:1193‐1211. doi:10.3233/JAD-180713 30412495

[62] Scelsi MA , Khan RR , Lorenzi M , et al. Genetic study of multimodal imaging Alzheimer's disease progression score implicates novel loci. Brain. 2018;141:2167‐2180. doi:10.1093/brain/awy141 29860282 PMC6022660

[63] Altmann A , Aksman LM , Oxtoby NP , et al. Towards cascading genetic risk in Alzheimer's disease. Brain. 2024;147:2680‐2690. doi:10.1093/brain/awae176 38820112 PMC11292901

[64] Gonzalez MC , Ashton NJ , Gomes BF , et al. Association of plasma p‐tau181 and p‐tau231 concentrations with cognitive decline in patients with probable dementia with Lewy bodies. JAMA Neurol. 2022;79:32‐37. doi:10.1001/jamaneurol.2021.4222 34807233 PMC8609462

[65] Diaz‐Galvan P , Przybelski SA , Algeciras‐Schimnich A , et al. Plasma biomarkers of Alzheimer's disease in the continuum of dementia with Lewy bodies. Alzheimers Dement. 2024;20:2485‐2496. doi:10.1002/alz.13653 38329197 PMC11032523

[66] McKeith IG , Ballard CG , Perry RH , et al. Prospective validation of consensus criteria for the diagnosis of dementia with Lewy bodies. Neurology. 2000;54:1050‐1058. doi:10.1212/WNL.54.5.1050 10720273

[67] Mattsson N , Rosén E , Hansson O , et al. Age and diagnostic performance of Alzheimer disease CSF biomarkers. Neurology. 2012;78:468‐476. doi:10.1212/WNL.0b013e3182477eed 22302554 PMC3280049

[68] Lewis ACF , Green RC . Polygenic risk scores in the clinic: new perspectives needed on familiar ethical issues. Genome Med. 2021;13:14. doi:10.1186/s13073-021-00829-7 33509269 PMC7844961

[69] Mielke MM , Dage JL , Frank RD , et al. Performance of plasma phosphorylated tau 181 and 217 in the community. Nat Med. 2022;28:1398‐1405. doi:10.1038/s41591-022-01822-2 35618838 PMC9329262

[70] Andrews SJ , Renton AE , Fulton‐Howard B , Podlesny‐Drabiniok A , Marcora E , Goate AM . The complex genetic architecture of Alzheimer's disease: novel insights and future directions. EBioMedicine. 2023;90:104511. doi:10.1016/j.ebiom.2023.104511 36907103 PMC10024184

[71] Belloy ME , Andrews SJ , Le Guen Y , et al. APOE genotype and Alzheimer disease risk across age, sex, and population ancestry. JAMA Neurol. 2023;80:1284‐1294. doi:10.1001/jamaneurol.2023.3599 37930705 PMC10628838

[72] Martin AR , Kanai M , Kamatani Y , Okada Y , Neale BM , Daly MJ . Clinical use of current polygenic risk scores may exacerbate health disparities. Nat Genet. 2019;51:584‐591. doi:10.1038/s41588-019-0379-x 30926966 PMC6563838

[73] Duncan L , Shen H , Gelaye B , et al. Analysis of polygenic risk score usage and performance in diverse human populations. Nat Commun. 2019;10:3328. doi:10.1038/s41467-019-11112-0 31346163 PMC6658471

[74] Jung S‐H , Kim H‐R , Chun MY , et al. Transferability of Alzheimer disease polygenic risk score across populations and its association with Alzheimer disease‐related phenotypes. JAMA Netw Open. 2022;5:e2247162. doi:10.1001/jamanetworkopen.2022.47162 36520433 PMC9856322

[75] Yang H‐S , Teng L , Kang D , et al. Cell‐type‐specific Alzheimer's disease polygenic risk scores are associated with distinct disease processes in Alzheimer's disease. Nat Commun. 2023;14:7659. doi:10.1038/s41467-023-43132-2 38036535 PMC10689816

[76] Hampel H , O'Bryant SE , Molinuevo JL , et al. Blood‐based biomarkers for Alzheimer disease: mapping the road to the clinic. Nat Rev Neurol. 2018;14:639‐652. doi:10.1038/s41582-018-0079-7 30297701 PMC6211654

