# Supplementary material for: Polygenic risk discriminates Lewy body dementia from Alzheimer's disease
Source: Alzheimers Dement. 2025 Jan 24;21(2):e14381. doi: 10.1002/alz.14381 (PMC11848393; doi:10.1002/alz.14381)
Supplement: Supplementary file 1 — Supporting Information [file ALZ-21-e14381-s001.docx]

**Supplementary material**

Polygenic risk discriminates Lewy body dementia from Alzheimer’s disease.

A McKeever ^a, b^, P Swann ^a^, M Malpetti ^c, d^, P C Donaghy ^e^, A Thomas ^e^, Elijah Mak ^a^, Stephen F Carter ^a^, Jerry HK Tan ^a^, Young T Hong ^c,f^, Tim D Fryer ^c,f^, A Heslegrave ^g,h,^, H Zetterberg  ^g, h, i, j, k, l^, L Su ^a, m^, L Chouliaras ^a, n^, J B Rowe ^c, d^ , J T O’Brien ^a, b, d^

**Sample collection and processing for genetic material and plasma biomarkers.**

Blood samples were collected by venepuncture in ETDA tubes, centrifuged, and stored at −70°C. Samples were thawed on wet ice, centrifuged at 500×g for 5 min at 4°C, and diluted 1:4.

DNA was isolated from buffy coat using Qiagen DNA isolation kits. DNA concentrations of 50ng/μL were used for the microarray. Genotyping was performed at two sites (UCL Genomics and Cambridge Genomics Services) using Illumina OmniExpress-24 v1.3 [1], to generate files in PLINK format.

Using established quality control procedures and methods to calculate polygenic scores, in PLINK 1.9 [3], we removed samples with genotype call rate <98% (--mind 0.02, n=0), outliers for heterozygosity (>3SD below mean, n=1), as well as variants with SNP missingness >5% (--geno 0.05, 38602 variants removed), failing Hardy-Weinberg equilibrium (--hwe 1e-6, 20970 variants removed), or with minor allele frequency <0.01 (--maf 0.01, 50530 variants removed). Linkage disequilibrium pruning was performed (--indep-pairwise 200 50 0.25) and closely related individuals were removed (threshold between second- and third-degree relatives applied, --king-cutoff 0.0884, n=1). After merging with 1000 Genomes reference data, genetic ancestry was determined using multidimensional scaling (MDS) and outliers for the first two MDS components defining European ancestry were excluded (n=2). Imputation was performed using the Michigan imputation server [4] and Haplotype Reference Consortium 1.1 reference panel [5]. Post imputation QC included removing variants that were non-biallelic, duplicates and those with poor imputation quality (R^2^ ≤ 0.3) [4]. The final quality-controlled data set included 167 individuals and 8,034,915 variants. After clumping and thresholding at p values <0.1, <1x10^-5^ and <5x10^-8^, the number of SNPs included in each PRS is shown in Supplementary Table 1.

Plasma biomarkers p-tau181, Aβ_42,_ Aβ_40_, GFAP and NfL, were quantified at the UK Dementia Research Institute biomarker laboratory as described previously [6]. Using the same ETDA tubes as those used for isolating genetic material, the Quanterix Simoa assay for p-tau181 V2 and﻿ Quanterix Simoa Human Neurology 4- Plex E assay for Aβ_40_, Aβ_42_, GFAP and NfL were performed using Simoa- HD1 as per the manufacturer’s protocol. Samples were analysed with the same batch of reagents. Two samples of known high and low concentrations were used for quality control. The mean percentage coefficients of variation were p-tau181 6.29%, Aβ_42_ 3.06%, Aβ_40_ 2.55%, GFAP 4.12%, NfL 4.08%. One participant (in the Control group) with logGFAP 5.9 standard deviations from sample mean (GFAP 6084pg/mL) was considered an outlier and removed from analysis of the plasma biomarker panel (Supplementary Table 4).

**Supplementary Table 1**. Number of SNPs included in AD-PRS

| p value threshold (pT) | n SNPs | |
| --- | --- | --- |
|  | AD-PRS including *APOE* | AD-PRS excluding *APOE* (AD-PRS_no_*_APOE_)* |
| 0.1 | 106,202 | 105,988 |
| 1x10^-5^ | 145 | 77 |
| 5x10^-8^ | 64 | 18 |

**Supplementary Table 1 abbreviations:**

AD-PRS = Alzheimer’s disease polygenic risk score

SNP = single nucleotide polymorphism

**Supplementary Figure 1.** Boxplots of AD-PRS_no_*_APOE_* using pT < 5x10^-8^, pT <1x10^-5^, and pT <0.1.


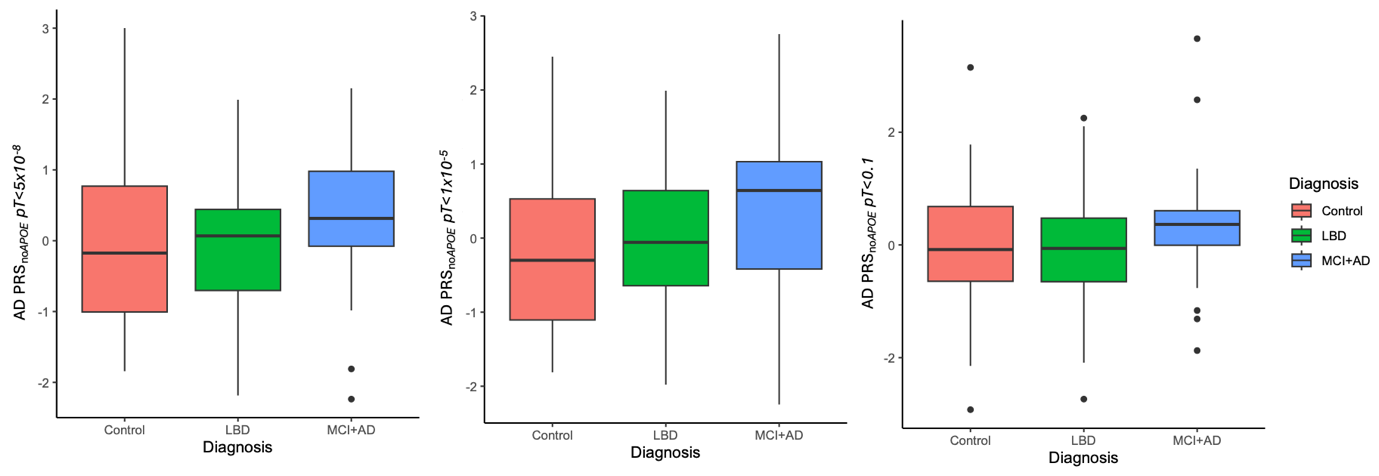


**Supplementary Figure 1** **abbreviations:**

MCI+/AD = PET Aβ positive mild cognitive impairment or Alzheimer’s disease dementia

LBD = Lewy body dementia

AD-PRS_no_*_APOE_* = Alzheimer’s disease polygenic risk score excluding *APOE* locus

pT = p value threshold

**Supplementary Table 2:** Diagnostic classification using AD-PRS_no_*_APOE_* (pT<5x10^-8^), *APOE*-RS and plasma p-tau181.

| ***Diagnosis*** | ***Model*** | ***OR [95% CI] (p value)*** | | | | | ***AUC [95% CI]*** | ***AICc*** |
| --- | --- | --- | --- | --- | --- | --- | --- | --- |
|  |  | *AD PRS*_noAPOE_ | *APOE-* RS | *p-tau181* | *Age* | *Sex* |  |  |
| Control vs MCI+/AD | *Polygenic risk* | 1.51  [0.964,  2.45]  (0.079) | **2.26****  **[1.37,**  **3.99]**  **(0.002)** | - | 1.06  [0.996,  1.14]  (0.076) | 1.34  [0.466,  4.03]  (0.595) | 0.758  [0.651,  0.865] | 102 |
|  | *p-tau181* | **-** | - | **3.41*****  **[1.85,**  **7.03]**  **(<0.001)** | 1.00  [0.932,  1.07]  (0.990) | 0.579  [0.180,  1.77]  (0.343) | 0.790  [0.684,  0.896] | 94.5 |
|  | *Polygenic risk +*  *p-tau181* | **1.72***  **[1.03,**  **3.03]**  **(0.046)** | 1.72  [0.993,  3.12]  (0.060) | **3.11****  **[1.60,**  **6.59]**  **(0.001)** | 1.03  [0.952,  1.11]  (0.490) | 0.742  [0.218,  2.48]  (0.626) | 0.812  [0.716,  0.907] | 92.0 |
| LBD  vs MCI+/AD | *Polygenic risk* | 1.71  [1.01,  3.00]  (0.050) | **1.67***  **[1.07,**  **2.66]**  **(0.026)** | **-** | 0.967  [0.904,  1.03]  (0.324) | 0.393  [0.142,  1.09]  (0.071) | 0.726  [0.603,  0.850] | 119 |
|  | *p-tau181* | **-** | - | **1.81***  **[1.13,**  **3.01]**  **(0.016)** | 0.938  [0.875,  1.00]  (0.057) | **0.333***  **[0.119,**  **0.925]**  **(0.034)** | 0.715  [0.612,  0.819] | 120 |
|  | *Polygenic risk +*  *p-tau181* | **1.73***  **[1.02,**  **3.04]**  **(0.046)** | 1.50  [0.95,  2.42]  (0.088) | **1.71***  **[1.04,**  **2.93]**  **(0.039)** | 0.947  [0.882,  1.01]  (0.124) | **0.336***  **[0.116,**  **0.962]**  **(0.042)** | 0.757  [0.654,  0.860] | 117 |
| LBD Aβ PET-  vs  LBD Aβ PET+ | *Polygenic risk* | 0.889  [0.396, 1.92]  (0.766) | **2.96* [1.29, 8.06]**  **(0.018)** | **-** | **1.13* [1.02, 1.28]**  **(0.029)** | 1.000  [0.159, 6.02]  (1.000) | 0.781 [0.647, 0.915] | 68.7 |
|  | *p-tau181* | **-** | - | 1.65  [0.867, 3.50] (0.153) | 1.08 [0.981, 1.21]  (0.123) | 0.515 [0.906, 2.47]  (0.419) | 0.736 [0.591, 0.881] | 71.7 |
|  | *Polygenic risk +*  *p-tau181* | 0.822 [0.350, 1.81]  (0.634) | **2.87* [1.20, 8.14]**  **(0.028)** | 1.63 [0.766, 3.76]  (0.220) | 1.12 [1.00, 1.26]  (0.053) | 0.883 [0.131, 5.80]  (0.895) | 0.796 [0.666, 0.927] | 69.7 |

**Supplementary Table 2 Abbreviations**

pT = p value threshold
MCI+/AD = PET Aβ positive mild cognitive impairment or Alzheimer’s disease dementia

LBD = Lewy body dementia

LBD PET Aβ- = Lewy body dementia, amyloid-β PET negative

LBD PET Aβ+ = Lewy body dementia, amyloid-β PET positive

*AD-PRS*_no_*_APOE_* =Alzheimer’s disease polygenic risk score excluding the *APOE* locus

*APOE-RS*= *APOE* risk score

AUC= area under receiver operating curve (for the model, including age and sex)

AICc= Akaike information criterion, with correction for sample size, quantifies trade-off between model fit and parsimony, Δ≥-2 indicates significant improvement

* Significant difference between groups p<0.001 (***), p<0.01 (**), p<0.05 (*).

All models include age and sex (Female=1, Male=2) as control covariates.

**Supplementary Table 3.** Diagnostic classification using AD-PRS_no_*_APOE_* (pT<1x10^-5^), *APOE*-RS and plasma p-tau181.

| ***Diagnosis*** | ***Model*** | ***OR [95% CI] (p value)*** | | | | | ***AUC***  ***[95% CI]*** | ***AICc*** |
| --- | --- | --- | --- | --- | --- | --- | --- | --- |
|  |  | AD-PRS_no_*_APOE_* | *APOE*-RS | p-tau181 | Age | Sex |  |  |
| Control vs MCI+/AD | *Polygenic risk* | **1.72***  **[1.07, 2.90]**  **(0.030)** | **2.21****  **[1.33, 3.94]**  **(0.004)** | - | 1.07  [0.999, 1.15]  (0.062) | 1.27  [0.435, 3.90]  (0.663) | 0.772  [0.666, 0.878] | 99.6 |
|  | *p-tau181* | **-** | - | **3.41*****  **[1.85, 7.03] (<0.001)** | 1.00  [0.932, 1.07]  0.990 | 0.579  [0.180, 1.77]  (0.343) | 0.790  [0.684-0.896] | 94.5 |
|  | *Polygenic risk +*  *p-tau181* | **1.95***  **[1.10, 3.51]**  **(0.018)** | 1.68  [0.948, 3.09]  (0.083) | **3.13****  **[1.61, 6.74]**  **(0.002)** | 1.03  [0.955, 1.11]  (0.445) | 0.706  [0.200, 2.42]  (0.579) | 0.832  [0.745-0.920] | 90.0 |
| LBD  vs MCI+/AD | *Polygenic risk* | 1.38  [0.858, 2.28] (0.195) | **1.65* [1.06, 2.62] (0.029)** |  | 0.969 [0.907, 1.04] (0.355) | 0.399  [0.146, 1.10] (0.073) | 0.702 [0.576,  0.827 | 122 |
|  | *p-tau181* | **-** | - | **1.81***  **[1.13, 3.01]**  **(0.016)** | 0.938  [0.875, 1.00]  (0.057) | **0.333***  **[0.119, 0.925] (0.034)** | 0.715  [0.612,  0.819] | 120 |
|  | *Polygenic risk +*  *p-tau181* | 1.33  [0.825, 2.20] (0.249) | 1.49  [0.944, 2.40]  (0.089) | 1.64 [1.01, 2.77]  (0.051) | 0.951 [0.886, 1.02]  (0.147) | **0.352* [0.124, 0.992]**  **(0.047)** | 0.736  [0.631, 0.842] | 120 |
| LBD Aβ PET-  vs  LBD Aβ PET+ | *Polygenic risk* | 0.885  [0.433, 1.77] (0.729) | **3.04* [1.34, 8.18] (0.015)** | - | **1.13* [1.02, 1.27] (0.029)** | 1.05  [0.170, 6.21]  (0.955) | 0.782 [0.650, 0.915] | 68.7 |
|  | *p-tau181* | **-** | - | 1.65  [0.867, 3.50] (0.153) | 1.08 [0.981, 1.21]  (0.123) | 0.515 [0.906, 2.47]  (0.419) | 0.736 [0.591, 0.881] | 71.7 |
|  | *Polygenic risk +*  *p-tau181* | 0.747  [0.329, 1.59] (0.459) | **3.01* [1.27 8.51]**  **(0.021)** | 1.766 [0.792, 4.372]  (0.186) | 1.12 [1.00, 1.26] (0.057) | 0.954 [0.146, 6.12]  (0.959) | 0.809 [0.684, 0.933] | 69.4 |

**Supplementary Table 3 Abbreviations**

pT = p value threshold
MCI+/AD = PET Aβ positive mild cognitive impairment or Alzheimer’s disease dementia

LBD = Lewy body dementia

LBD PET Aβ- = Lewy body dementia, amyloid-β PET negative

LBD PET Aβ+ = Lewy body dementia, amyloid-β PET positive

*AD-PRS*_no_*_APOE_* =Alzheimer’s disease polygenic risk score excluding the *APOE* locus

*APOE-RS*= *APOE* risk score

AUC= area under receiver operating curve (for the model, including age and sex)

AICc= Akaike information criterion, with correction for sample size, quantifies trade-off between model fit and parsimony, Δ≥-2 indicates significant improvement

* Significant difference between groups p<0.001 (***), p<0.01 (**), p<0.05 (*).

All models include age and sex (Female=1, Male=2) as control covariates.

**Supplementary Table 4.** Diagnostic classification using AD-PRS_no_*_APOE_* (pT<0.1), *APOE*-RS and a panel of plasma biomarkers. Excluding 1 outlier from the Control group (Control n=56, MCI+/AD n=27, LBD n=83).

| ***Diagnosis*** | ***Model*** | ***Odds Ratio [95% CI] (p value)*** | | | | | | | | ***AUC***  ***[95% CI]*** | ***AICc*** |
| --- | --- | --- | --- | --- | --- | --- | --- | --- | --- | --- | --- |
|  |  | *AD-PRS_noAPOE_* | *APOE-RS* | *p-tau181* | Aβ_42/40_ | *GFAP* | *NfL* | *Age* | *Sex* |  |  |
| Control  vs MCI+  /AD | Plasma bio-markers | **-** | **-** | **3.19****  **[1.56, 7.34]**  **(0.003)** | **0.476***  **[0.220, 0.940]**  **(0.044)** | 1.46 [0.672, 3.26]  (0.345) | 0.790 [0.318, 1.06]  (0.601) | 1.01 [0.919, 1.10]  (0.897) | 0.690 [0.186, 2.45]  (0.566) | 0.818  [0.723, 0.912] | 94.4 |
|  | PRS +  plasma bio-markers | 1.70 [0.97, 3.27]  (0.08) | 1.64 [0.91, 3.09]  (0.11) | **3.15** [1.46, 7.61]**  **(0.006)** | 0.494 [0.20, 1.030]  (0.069) | 1.13 [0.477, 2.67]  (0.795) | 0.884 [0.342, 2.25]  (0.898) | 1.01 [0.921,1.12]  (0.783) | 0.783 [0.200, 2.97]  (0.718) | 0.841  [0.754, 0.929] | 94.2 |
|  | Parsimonious model | 1.74  [1.02, 3.31] (0.059) | 1.70 [0.987, 3.08] (0.064) | **3.15*****  **[1.69, 6.57]**  **(<0.001)** | 0.487 [0.223, 0.988]  (0.054) |  |  |  |  | 0.833  [0.741, 0.925] | 85.0 |
| LBD  vs MCI+  /AD | Plasma bio-markers | **-** | **-** | **2.06** [1.23, 3.62]**  **(0.008)** | 0.646 [0.368, 1.10]  (0.108) | 1.78 [0.812, 4.12]  (0.161) | 0.496 [0.119, 1.11]  (0.108) | 0.945 [0.873,1.02]  (0.154) | **0.329* [0.109, 0.976]**  **(0.045)** | 0.743  [0.641,0.846] | 120 |
|  | PRS +  plasma bio-markers | **1.84* [1.06, 3.43]**  **(0.038)** | 1.66 [1.00, 2.82]  (0.053) | **2.08* [1.21, 3.82]**  **(0.012)** | 0.580 [0.321, 1.04]  (0.062) | 1.44 [0.639, 3.37]  (0.387) | 0.512 [0.206, 1.15]  (0.122) | 0.947 [0.870, 1.03]  (0.203) | 0.369 [0.117, 1.14]  (0.083) | 0.784  [0.682,0.885] | 118 |
|  | Parsimonious model | **1.84* [1.07, 3.33]**  **(0.031)** | **1.82***  **[1.12, 3.05]**  **(0.018)** | **1.96* [1.15,3.58]**  **(0.019)** | 0.593 [0.335, 1.04]  (0.065) |  | **0.537* [0.280, 0.958]**  **(0.045)** |  | 0.400  [0.134, 1.19]  (0.097) | 0.776  [0.676,0.877] | 115 |
| LBD  PET Aβ-  vs  LBD  PET Aβ+ | Plasma bio-markers | **-** | **-** | 1.65  [0.859, 3.55]  (0.159) | 0.960 [0.504, 1.74]  (0.892) | 2.30 [0.749, 8.08]  (0.161) | 0.757 [0.254, 2.15]  (0.601) | 1.08 [0.960, 1.22] (0.205) | 0.571 [0.091, 3.04]  (0.520) | 0.761 [0.623,0.899] | 76.6 |
|  | PRS +  plasma bio-markers | 0.616 [0.276, 1.30]  (0.208) | **2.71* [1.11, 7.69]**  **(0.040)** | 1.60  [0.740, 3.81]  (0.257) | 0.840 [0.408, 1.69]  (0.625) | 1.46 [0.435, 5.45]  (0.548) | 0.901 [0.284, 2.73]  (0.854) | 1.11 [0.981, 1.28]  (0.109) | 0.750 [0.107, 5.09]  (0.763) | 0.841 [0.727,0.955] | 75.2 |
|  | Parsimonious model | 0.584 [0.269, 1.19]  (0.145) | **2.87***  **[1.29, 7.48]**  **(0.017)** |  |  |  |  | **1.15* [1.04, 1.29] (0.014)** |  | 0.790 [0.663,0.917] | 64.2 |

**Supplementary Table 4 Abbreviations**

pT = p value threshold
MCI+/AD = PET Aβ positive mild cognitive impairment or Alzheimer’s disease dementia

LBD = Lewy body dementia

LBD PET Aβ- = Lewy body dementia, amyloid-β PET negative

LBD PET Aβ+ = Lewy body dementia, amyloid-β PET positive

*AD-PRS*_no_*_APOE_* =Alzheimer’s disease polygenic risk score excluding the *APOE* locus

*APOE-RS*= *APOE* risk score

p-tau181= plasma phosphorylated tau at threonine-181

Aβ_42/40_ = ratio of Aβ_42_ to Aβ_40_

GFAP = glial fibrillar acidic protein

NfL = neurofilament light

AUC= area under receiver operating curve (for the model, including age and sex)

AICc= Akaike information criterion, with correction for sample size, quantifies trade-off between model fit and parsimony, Δ≥-2 indicates significant improvement

* Significant difference between groups p<0.001 (***), p<0.01 (**), p<0.05 (*).

All models include age and sex (Female=1, Male=2) as control covariates.

**Supplementary References:**

[1] Gibbons E, Rongve A, Rojas I de, Shadrin A, Westra K, Baumgartner A, et al. Identification of a sex-specific genetic signature in dementia with Lewy bodies: a meta-analysis of genome-wide association studies. MedRxiv 2022:2022.11.22.22282597. doi:10.1101/2022.11.22.22282597.

[2] Leonenko G, Baker E, Stevenson-Hoare J, Sierksma A, Fiers M, Williams J, et al. Identifying individuals with high risk of Alzheimer’s disease using polygenic risk scores. Nat Commun 2021;12:4506. doi:10.1038/s41467-021-24082-z.

[3] Choi SW, Mak TS-H, O’Reilly PF. Tutorial: a guide to performing polygenic risk score analyses. Nat Protoc 2020;15:2759–72. doi:10.1038/s41596-020-0353-1.

[4] Das S, Forer L, Schönherr S, Sidore C, Locke AE, Kwong A, et al. Next-generation genotype imputation service and methods. Nat Genet 2016;48:1284–7. doi:10.1038/ng.3656.

[5] McCarthy S, Das S, Kretzschmar W, Delaneau O, Wood AR, Teumer A, et al. A reference panel of 64,976 haplotypes for genotype imputation. Nat Genet 2016;48:1279–83. doi:10.1038/ng.3643.

[6] Chouliaras L, Thomas A, Malpetti M, Donaghy P, Kane J, Mak E, et al. Differential levels of plasma biomarkers of neurodegeneration in Lewy body dementia, Alzheimer’s disease, frontotemporal dementia and progressive supranuclear palsy. J Neurol Neurosurg Psychiatry 2022;93:651–8. doi:10.1136/jnnp-2021-327788.
